# S. Padmavati: The Trailblazing Pioneer of Cardiovascular Medicine in India

**DOI:** 10.7759/cureus.73616

**Published:** 2024-11-13

**Authors:** Manjari V, Kalaivanan K, Abarna B, Harishma K, Nithyashree M

**Affiliations:** 1 Department of Siddha Toxicology, National Institute of Siddha, Chennai, IND; 2 Department of Indian Medicine and Homeopathy, Government Primary Health Center, Thiruvannamalai, IND; 3 Department of Siddha Medicine, Nandha Siddha Medical College and Hospital, Erode, IND

**Keywords:** cardiologist, cardiovascular medicine, heart diseases, historical vignette, padmavathi iyer

## Abstract

Dr. S. Padmavathi Iyer is a distinguished cardiologist recognized for her expertise in cardiovascular medicine. With a focus on advanced cardiac care, she has contributed significantly to the diagnosis, treatment, and management of heart diseases. Her work integrates cutting-edge techniques with a patient-centered approach, aiming to improve outcomes for individuals with complex cardiovascular conditions. Her research and clinical practice emphasize the importance of personalized care and innovative solutions in cardiology.

## Introduction and background

**Figure 1:**
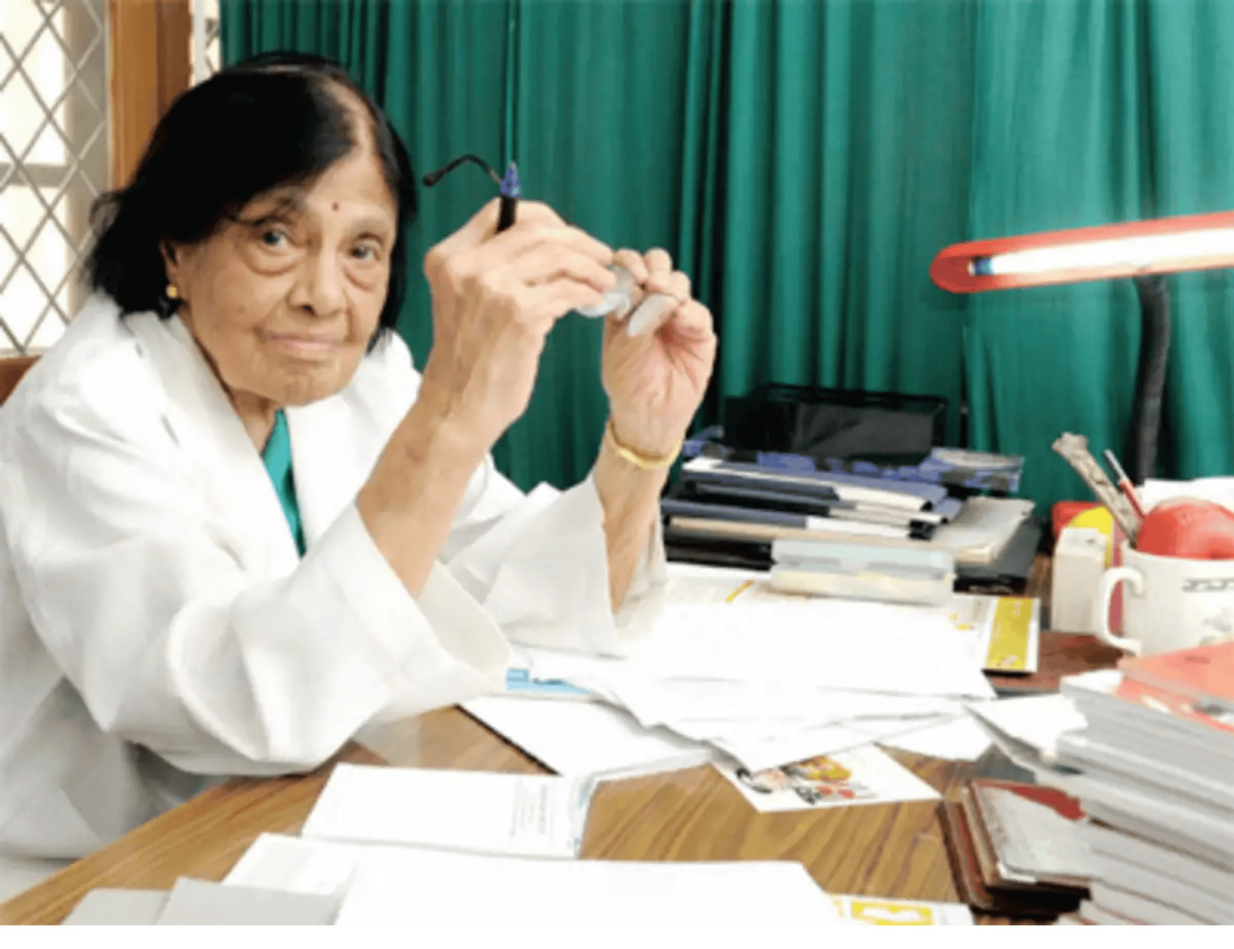
Dr. Padmavathi Iyer Source: [1] Copyright: Public Domain

Dr. S. Padmavati, also known as "Paddy," is an eminent figure in the history of cardiovascular medicine, not only in India but throughout the Asian peninsula (Figure 1) [1,2]. Her contributions to the field of cardiovascular disease (CVD) epidemiology, prevention, and research have been irreversibly impactful, as she is a prominent cardiologist. The journey of Dr. Padmavati Iyer, from her early origins in Burma to her revolutionary work in India, is a monument to her steadfast commitment to public health and medical science [3].

## Review

Personal life

Biographical Overview: Early Life and Education

S. Padmavati was born in 1917 in Burma (currently Myanmar), and she showed exceptional scholastic talent from a young age. Being the first female student to receive a medical degree from Rangoon Medical College, she made history. Sadly, her promising start was cut short by the chaos of World War II. When Japan invaded Burma in 1942, Dr. Padmavati, her mother, and her four sisters went to Coimbatore, Tamil Nadu, leaving their male relatives behind. Her father and brothers were lost to her until the end of the war [4].

Despite the difficulties she encountered, Dr. Padmavati continued her medical education overseas. The Royal College of Physicians in Edinburgh and the Royal College of Physicians in London awarded her fellowships. She next traveled to Sweden to continue her medical education, where she trained at the Sodersjukhuset Hospital in Stockholm under Drs. Gustav Nylin and Gunnat Bjorck [4].

Professional life

Advanced Training in the USA: Under the Guidance of Legends

Dr. Padmavati Iyer's interest led her to the United States, where she studied under Dr. Helen Taussig, a pioneering pediatric cardiologist best recognized for her groundbreaking research on congenital heart problems at the prestigious Johns Hopkins Hospital. She continued to improve her talent at Harvard Medical under Dr. Paul Dudley White [5].

A Return to India: Pioneering Cardiology in the Subcontinent

Dr. Padmavati brought a wealth of knowledge and experience with her when she returned to India in 1954. At Lady Harding Medical College (LHMC), Delhi, she was recruited as a lecturer and quickly promoted to the rank of professor and head of the department of medicine. In 1954, she was the founder of the first cardiac catheterization laboratory in North India, marking a significant advancement in the field of cardiology in the area [6].

Dr. Padmavati also had a wide range of significant research interests. Her epidemiological studies on hypertension, ischemic heart disease, pulmonary heart disease, and rheumatic fever continue to have an impact and are regularly referenced in current studies. Her passion for clinical innovation matched her devotion to research; as a result of her leadership, Maulana Azad Medical College (MAMC) established the first DM courses in cardiology and other super-specialties, the first coronary care unit in India, and the first coronary care van [6].

Leadership and Legacy: Building Institutions and Influencing Public Health

Over the course of her remarkable career, Dr. Padmavati occupied a number of leadership roles that influenced the direction of cardiology in India. She was a key member of governing bodies such as the Indian Council of Medical Research, All India Institute of Medical Sciences, and Jayadeva Institute of Cardiology in Bangalore. She held the position of president of both the Cardiological Society of India and the National Academy of Medical Sciences. Then she became the dean of the Institute [7].

Dr. Padmavati established the All India Heart Foundation in 1962 with the goal of improving heart health in all of India. She founded the National Heart Institute in 1981 and turned it into a state-of-the-art tertiary care facility with an emphasis on patient care, research, and community outreach under its auspices. Her vision and work laid the foundation for contemporary cardiology in India [7].

Scientific Research Contribution: Cardiovascular Medicine

Dr. Padmavati Iyer conducted clinical investigations and published over 300 research articles on preventive cardiovascular medicine [8]. She contributed extensively to the field of RF and RHD, cor pulmonale, and the epidemiology of hypertension and ischemic heart disease. She played a key role in the national infrastructure for RHD control and prophylaxis. Her work was published in reputed journals and quoted in many textbooks [9]. As an active proponent of government intervention in regulating cardiovascular disease risk factors. She conducted the first epidemiological survey on the incidence of ischemic heart disease in various parts of India, which was published in Circulation in 1962 [8].

Advocacy for Preventive Medicine: A Visionary Approach

In addition to being a researcher and clinician, Dr. Padmavati was a fervent supporter of cardiovascular preventative medicine. She was a strong advocate for government legislation to address these issues, particularly the control of fast food and cigarettes, and concentrated a large portion of her research on lifestyle-related CVD risks. She had a key role in steering the International Society of Cardiology (ISC) toward epidemiological research and prevention through her early involvement with the organization. This resulted in the establishment of the ISC Council on Epidemiology during the World Congress in Delhi in 1966 [10].

Honors and Recognition: A Life of Service

Many honors were bestowed upon Dr. Padmavati for her groundbreaking work. Her achievement of being the first female cardiologist in India was groundbreaking in and of itself. She received the Padma Bhushan in 1967 and the Padma Vibhushan in 1992, two of the highest civilian awards given by the Indian government, in appreciation for her achievements. Other notable honors were the Lifetime Award from the Cardiological Society of India (2012), the Sivananda Eminent Citizen Award (2012), the BC Roy Award, the Kamla Menon Research Award, the Harvard Medical International Award, and the Antonio Samia Oration of the Asia Pacific Society of Cardiology (2005) [11].

A Life of Dedication: Remembering Dr. Padmavathi

Dr. Padmavati was renowned for her humility and devotion to her patients and students, even in the face of her professional obligations. Even in her later years, she remained involved in the medical community, serving as a mentor to aspiring physicians and making contributions to medical research. 2020 saw the death of Dr. Padmavati, who was 103 years old. She left behind a legacy of distinction in cardiology, a dedication to medical education, and a pioneering attitude that served as an inspiration to many women in the medical field. Her life serves as an example of the influence one person may have on a whole field as well as public health in general [8,9].

## Conclusions

Dr. S. Padmavati’s remarkable life teaches valuable lessons: persevere through challenges, break barriers, strive for excellence through continuous learning, lead with vision and compassion, and dedicate yourself to helping others. Her pioneering spirit, innovative healthcare approaches, and advocacy for preventive medicine inspire women in medicine, healthcare professionals, and anyone seeking to make a positive impact. As India's first woman cardiologist, Dr. Padmavati's legacy demonstrates that age is not a limit to achieving greatness, leaving behind a testament to the profound impact one individual can have on medicine and public health.
